# Ferroptosis and its Potential Determinant Role in Myocardial Susceptibility to Ischemia/Reperfusion Injury in Diabetes

**DOI:** 10.31083/j.rcm2510360

**Published:** 2024-10-09

**Authors:** Dongcheng Zhou, Yuhui Yang, Ronghui Han, Jianfeng He, Danyong Liu, Weiyi Xia, Yin Cai, Bartłomiej Perek, Zhengyuan Xia

**Affiliations:** ^1^Department of Anesthesiology, Affiliated Hospital of Guangdong Medical University, 524000 Zhanjiang, Guangdong, China; ^2^Cardiac Surgery and Transplantology Department, Poznan University of Medical Sciences, 61-701 Poznan, Poland; ^3^Department of Health Technology and Informatics, The Hong Kong Polytechnic University, Hong Kong SAR, China; ^4^Doctoral Training Platform for Research and Translation, 431913 Zhongxiang, Hubei, China

**Keywords:** ferroptosis, myocardial ischemia/reperfusion-related injury, diabetic cardiomyopathy, oxidative stress

## Abstract

Myocardial ischemia/reperfusion injury (MIRI) is a major cause of cardiac death particularly in patients with diabetes. When the coronary artery is partially or completely blocked, restoration of blood perfusion can normally be achieved within a certain time due to the development of advanced techniques such as percutaneous coronary intervention (PCI) and coronary artery bypass grafting (CABG) surgery. However, cardiac tissue injury may aggravate progressively even after the ischemic myocardium is restored to normal perfusion. MIRI is often associated with various forms of cell death, including apoptosis, autophagy, programmed necrosis, pyroptosis, and ferroptosis, among others. Ferroptosis is known as iron-dependent cell death that is distinct from other programmed modes of cell death. Ferroptosis is under constitutive control by glutathione peroxidase 4 (GPX4), and the reduction of GPX4 may result in ferroptosis even if iron homeostasis is physiologically maintained. The essences of ferroptosis are substantial iron accumulation and lipid peroxidation that trigger cell death. Under impaired antioxidant system, cellular reactive oxygen species (ROS) accumulation leads to lipid peroxidation which consequently results in ferroptosis. Ferroptosis shares a few common features with several types of cell death and interplays with various forms of cell death such as autophagy and apoptosis in the development of cardiovascular diseases. More and more recent studies have demonstrated that ferroptosis plays an important role in MIRI. However, few studies have addressed the relative importance of ferroptosis in MIRI relative to other forms of cell deaths. In this review, we summarized the basic aspects and advances regarding the molecular pathogenesis of ferroptosis, evaluated its role in MIRI, and propose that the levels of ferroptosis may function as a major determinant of myocardial susceptibility to ischemia/reperfusion injury (IRI) in general and of the enhanced vulnerability to MIRI specifically in diabetes.

## 1. Introduction

Acute myocardial infarction (AMI) is the leading cause of death and disability worldwide. Early and rapid vascular re-canalization is the key to the treatment of AMI patients, which can effectively improve patient prognosis [1]. Myocardial ischemia/reperfusion injury (MIRI) consists of two major events, namely ischemia and reperfusion, and the outcome results from various harmful cell damages. During the myocardial ischemia period, the blood perfusion of the heart is reduced or blocked, resulting in reduced oxygen supply of the heart, abnormal myocardial energy metabolism, and compromised heart function. This leads to a series of detrimental changes in myocardial ultrastructure, energy metabolism and cardiac function. Restoration of blood perfusion to the ischemic area of the myocardium may cause significant pathophysiological changes in cardiomyocytes and local vasculature in the reperfused area, which together can promote further tissue injury and even severe arrhythmias and lead to sudden death. However, the exact molecular mechanisms and pathways associated with MIRI are unknown and highly controversial. In recent years, programmed cell deaths, including apoptosis, autophagy, necroptosis, pyroptosis, and ferroptosis have all been shown to be attributable to myocardial loss during ischemia/reperfusion injury (IRI) [2]. Ferroptosis is a recently identified cell death caused by iron-dependent and reactive oxygen species (ROS)-induced lipid peroxidation [3]. Ferroptosis plays an important role in a number of ischemia reperfusion models. In particular, it plays a critical role in MIRI including MIRI in diabetes [2]. In this review, we will discuss the mechanisms of ferroptosis induction and how it may negatively impact on the outcomes of MIRI.

## 2. An Overview of Ferroptosis

In 2003, Dolma *et al*. [4] found that erastin induced a type of cell death which caused selective lethal damages to cancer cells in a different manner other than previously reported. The authors noted it as a nonapoptotic cell death process initiated by erastin. This kind of death did not demonstrate nuclear morphological changes, nor did there exist DNA fragmentation or caspase activation, and caspase inhibitors could not reverse this type of cell death. Subsequently, Yang *et al*. [5] and Yagoda *et al*. [6] found that this type of cell death could be inhibited by iron chelating agents, and also found that the compound RSL3 (which was known as a selective ferroptosis inducer later on), could lead to this type of cell death. In 2012, Dixon *et al*. [7] formally named this type of cell death - ferroptosis. However, ferroptosis-induced cell death is clearly distinct in cell morphology and function from other modes of programmed death, such as apoptosis, autophagy, and necrosis (Table 1, Ref. [2, 3]). Ferroptosis is associated with significant changes in mitochondrial morphology, including mitochondrial shrinkage, outer membrane rupture, and increased membrane density. From a biochemical perspective, intracellular glutathione (GSH) depletion and reduced glutathione peroxidase 4 (GPX4) activity are observed during the development of ferroptosis. Genetically, ferroptosis is a biological process regulated by multiple genes. Ferroptosis mainly involves genetic changes in iron regulation and lipid peroxidation, but the specific regulatory mechanisms need to be further investigated [8].

**Table 1.** **The features of ferroptosis, apoptosis, necroptosis and autophagy**.

	Ferroptosis	Apoptosis	Necroptosis	Autophagy
Predisposing factors	Iron accumulation	Gene regulation under normal physiological conditions	Pathological factors	Nutritional deficiency or hormonal induction
Morphological feature				
Membrane	No rupture or blistering of the plasma film	Plasma membrane blistering and cell aggregation	Rupture of the plasma membrane	No changes
Cytoplasm	Small mitochondria and increased mitochondrial membrane densities	Pseudopod contraction and cell volume reduction	Swelling of cytoplasmic organelles	Accumulation of double-membra autophagic vacuoles
Nucleus	Normal nuclear size non-cohesive chromatin	Reduction of nuclear volume chromatin condensation	Moderate chromatin condensation	Non-cohesive chromatin
Biochemical features	Iron accumulation and lipid peroxidation	DNA fragmentation	ATP depletion and release of DAMP	Increased lysosomal activity
Regulatory pathways	GPX4/GSH pathway, FSP1/CoQ10/NADPH pathway, DHODH-CoQH2pathway, GCH1/BH4 pathway	Caspase, Bcl-2, p53 mediated signaling pathway	RIP1/RIP3 related signaling pathway	mTOR, Beclin- 1 related signaling pathway
Key genes	*GPX4*, *SLC7A11*, *FSP1*, *GCH1*, *ACSL4*, *NCOA4*	*Caspase*, *Bcl-2*, *p53*, *Bax*, *Fas*	*RIP1*, *RIP3*	*mTOR*, *Beclin- 1*, *NRF2*, *LC3*, *ATG5*, *ATG7*
References	[2, 3]	[2]	[2]	[2]

Abbreviation used: GSH, glutathione; GPX4, glutathione peroxidase 4; FSP1, ferroptosis suppressor protein 1; CoQ10, coenzyme Q10; NADPH, nicotinamide adenine dinucleotide phosphate; DHODH, dihydroorotate dehydrogenase; GCH1, guanosine 5^′^-triphosphate cyclohydrolase 1; BH4, tetrahydrobiopterin; Bcl-2, members of the B cell lymphoma 2; RIP1, receptor interaction protein kinases 1; RIP3, receptor interaction protein kinases 3; mTOR, mammalian target of rapamycin; NRF2, nuclear factor-erythroid 2-related factor 2; SLC7A11, solute carrier family 7 member 11; ACSL4, acyl-CoA synthetase long-chain family member 4; NCOA4, nuclear receptor coactivator 4; Bax, Bcl-2 associated protein X; LC3, microtubule-associated protein 1 light chain 3; ATG5, autophagy-related 5; ATG7, autophagy-related 7; ATP, adenosine triphosphate; DAMP, damage-associated molecular patterns; CoQH2, ubiquinol; p53, tumor protein p53; Fas, factor-related apoptosis.

## 3. Prerequisites for Ferroptosis

**Fig. 1.**
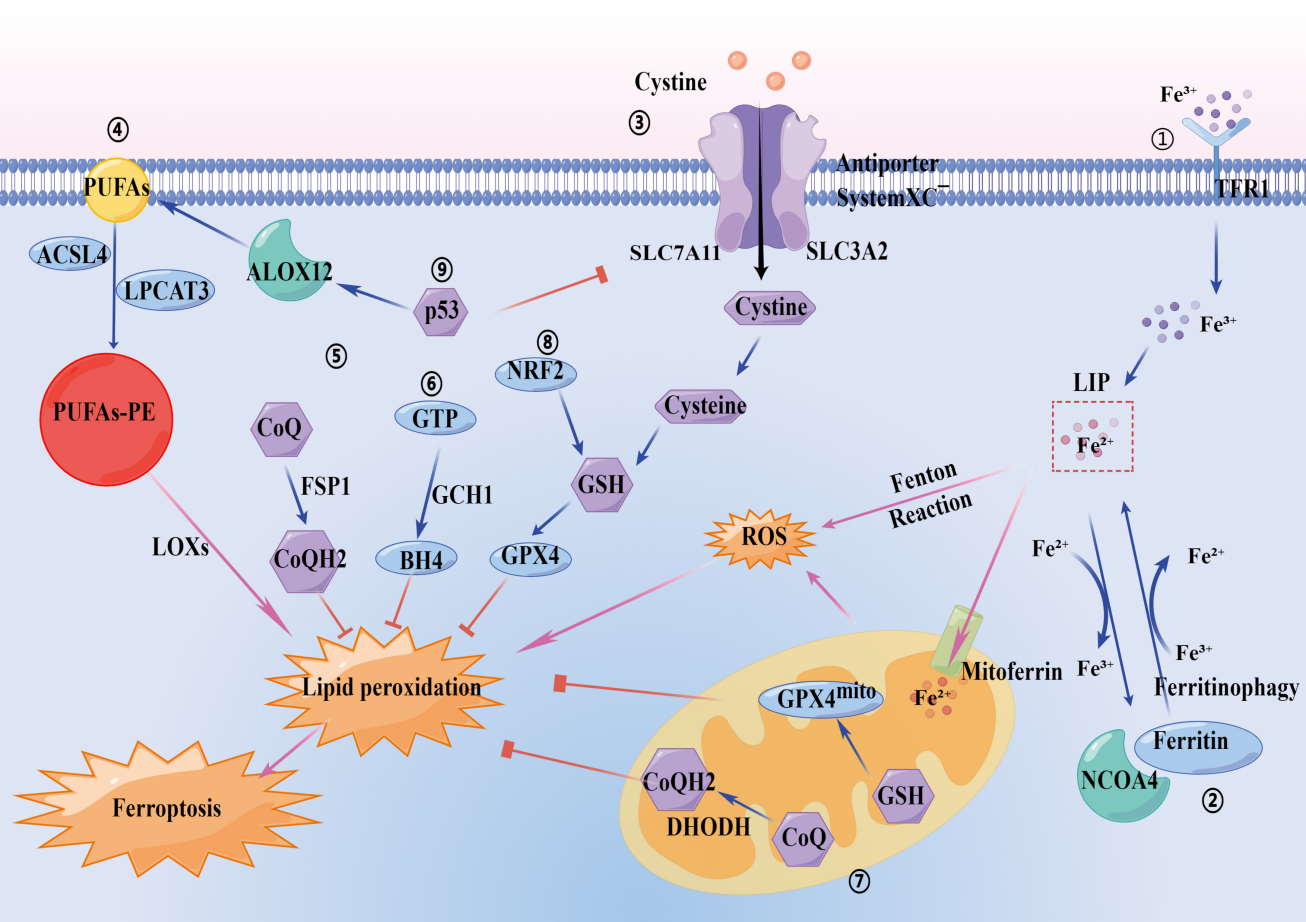
**Regulatory metabolic pathways of ferroptosis**. ① Iron metabolism pathway: Fe^3+^ is imported through transferrin receptor 1 (TFR1). Fe^3+^ is converted into Fe^2+^ and released into the cytoplasm. Fe^2+^ participates in the Fenton Reaction, producing lipid ROS and causing ferroptosis. ② Ferritinophagy: Ferritin stores iron and reduces Fe^2+^ to Fe^3+^, limiting the Fenton Reaction. NCOA4 binds ferritin mediating its autophagic degradation in a process called ferritinophagy. This mechanism promotes ferroptosis. ③ GPX4 pathway: Amino acid antiporter Systemxc-(composed by SLC3A2 and SLC7A11 subunits) mediates the exchange of extracellular cystine and intracellular glutamate. It absorbs extracellular cysteine to promote glutathione synthesis. Cystine inhibition triggers ferroptosis through GSH depletion. GPX4 can catalyze the reduction of lipid peroxides thus preventing ferroptosis. ④ Lipid metabolism pathway: PUFAs and phosphatidylethanolamine (PE) derived from lipid bilayers are metabolized by ACSL4 and LPCAT3 and then oxidized by LOXs to produce lipid peroxidation. ⑤ FSP1-CoQ10-NADPH pathway: FSP1 can reduce CoQ10 to CoQH2 which, in turn, blocks lipid peroxidation. ⑥ GCH1/BH4/DHFR pathway: GCH1-BH4 pathway, which acts independently of the GPX4 pathway to regulate ferroptosis by inhibiting lipid peroxidation. ⑦ Mitochondria pathway: Iron from LIP can be transported to the mitochondria via mitochondrial ferritin (mitoferrin) and stored in ferritin. Abnormal mitochondrial iron regulation causes a massive production of ROS, which disrupts lipid peroxidation and promotes cell ferroptosis. Mitochondrial GPX4 (GPX4mito) and DHODH constitute the two main defenses against mitochondrial lipid peroxidation. In mitochondria, GPX4 attenuates ferroptosis by reducing lipid peroxidation to lipid alcohols using GSH as its cofactor. In addition, DHODH, present on the outer surface of the inner mitochondrial membrane, reduces CoQ to CoQH2, which in turn reduces lipid peroxidation and thus inhibits ferroptosis. ⑧ NRF2 pathway: NRF2 can regulate GSH homeostasis upstream, which can reduce the reduction of lipid peroxides and prevent the cells from ferroptosis. ⑨ p53 pathway: p53 can be transcribed to inhibit SLC7A11 expression and promote ferroptosis. Abbreviation used: ROS, reactive oxygen species; NCOA4, nuclear receptor coactivator 4; GPX4, glutathione peroxidase 4; SLC3A2, solute carrier family 3 member 2; SLC7A11, solute carrier family 7 member 11; GSH, glutathione; ACSL4, long-chain acyl-CoA synthetase-4; LPCAT3, lysophosphatidylcholine acyltransferase 3; LOXs, lipoxygenase; FSP1, ferroptosis suppressor protein 1; CoQ10, coenzyme Q10; NADPH, nicotinamide adenine dinucleotide phosphate; CoQH2, ubiquinol; GCH1, guanosine 5^′^-triphosphate cyclohydrolase 1; BH4, tetrahydrobiopterin; DHFR, dihydrofolate reductase; LIP, labile iron pool; GPX4mito, mitochondrial GPX4; DHODH, dihydroorotate dehydrogenase; CoQ, coenzyme Q; NRF2, nuclear factor erythroid 2-related factor 2; PUFAs, polyunsaturated fatty acids; GTP, guanosine triphosphate; p53, tumor protein p53; ALOX12, arachidonate 12-lipoxygenase.

The key to the execution of ferroptosis include the disruption of iron metabolism as well as the peroxidation of polyunsaturated fatty acids (PUFAs). Iron, PUFAs and ROS play irreplaceable roles in cell survival. However, they are all lethal to cells in the event of metabolic disorders. As will be described in this section, PUFA peroxidation, iron metabolism and mitochondrial metabolism constitute the main prerequisites driving ferroptosis (Fig. 1).

### 3.1 Iron Metabolism

Iron is readily reduced to Fe^2+^ and then oxidized toFe^3+^. Iron is able to easily change its valence state, providing or accepting electrons. Therefore, iron is in a privileged position as a critical biochemical reaction in living organisms [9]. Fe^2+^ can react with hydrogen peroxide (H_2_O_2_) to produce an even stronger OH^–^ oxidation capacity, thus increasing the level of ROS in cells [10]. This chemical property also makes iron the main catalyst for the production of active radicals. Thus, Fe^2+^ is able to induce oxidative stress. This suggests that during the process of ferroptosis, labile iron levels increase. Thus, iron loading is an important marker of ferroptosis.

The main part of cellular iron is well protected in the active site of the enzyme and safely stored in ferritin. When iron deficiency occurs, ferritin releases small amounts of iron through autophagy. This process is known as ferritinophagy [11, 12]. Thus, autophagy can affect iron metabolism through autophagy regulation. This suggests a close link between autophagy and ferroptosis. Ferritinophagy is mainly regulated by nuclear receptor coactivator 4 (NCOA4), and NCOA4-mediated ferritinophagy causes ferritin degradation and subsequently promotes the release of labile iron, which is a key factor in ferroptosis [11, 12].

However, under normal circumstances, cells also contain a small fraction of unbound iron which is very unstable and thus is known as a “labile iron pool (LIP)” [13, 14]. LIP plays a major role in ROS-induced toxicity, as well as in redox signaling [15, 16]. In any case, LIP can interact with peroxides to produce highly active substances. These reactions are typically diffusion-controlled (Fig. 1). In conclusion, iron plays an important role in both oxidative stress and ferroptosis. Thus, regulation of iron metabolism and ferritinophagy are potential control points of ferroptosis.

### 3.2 Abnormal Lipid Metabolism

Lipid metabolism also plays an important role in determining cell sensitivity to ferroptosis. Lipid peroxides are considered a landmark event of ferroptosis [17]. The harm of lipid peroxidation is mainly reflected in its leading to the oxidative degradation of PUFAs and phosphatidylethanolamine (PE) [18].

PUFAs change the molecular structure, disrupt the fluidity and stability of the cell membrane structure, increase cell membrane permeability, and subsequently, cells are prone to rupture and death. PUFAs have a high affinity with the free radical, and the hydrogen atoms between its double bonds are easily oxidized [18]. Lipid peroxyl radicals capture hydrogen atoms from other PUFAs to form ROS and H_2_O_2_. It can be constantly involved in the PUFAs oxidation process. The lipid peroxidation reaction of PUFAs has a cascade signature, which subsequently disrupts the membranes and promotes the progression of ferroptosis. It has been shown that lysophosphatidylcholine acyltransferase 3 (LPCAT3) [18] and long-chain acyl-CoA synthetase-4 (ACSL4) [19] are involved in PUFAs lipid peroxidation on cell membranes. Alternatively, the PUFAs peroxidation process is also regulated by lipoxygenase (LOXs) and GPX4 activity [20].

However, unlike PUFAs, the affinity of PE for free radicals is not high. Before undergoing lipid peroxidation, PE needs ACSL4 and LPCAT3 to form oxidation sites. Firstly, arachidonic acid (AA) and adrenic acid (AdA) synthesize AA-CoA and AdA-CoA by ACSL4. Then, under LPCAT3 catalysis, AA/AdA-CoA forms PE-AA/AdA with PE. Ultimately, its uncontrolled accumulation leads to ferroptosis (Fig. 1).

### 3.3 Mitochondrial Metabolism

Cellular metabolism is crucial for ferroptosis since lipid peroxides are mainly produced by various steps of cellular metabolism. Growing evidence shows that different cellular metabolic processes, including lipid metabolism and amino acid metabolism (especially cysteine and glutamine) can contribute to the development of ferroptosis [21, 22]. Considering the central role of mitochondria in the regulation of oxidative metabolism and cell death, it is highly possible that mitochondria are involved in the regulation of ferroptosis [7]. Indeed, a large number of studies have shown that multiple metabolic activities of mitochondria have an impact on ferroptosis [23, 24, 25].

Under electron microscopy, the most striking feature of cells undergoing ferroptosis is the altered mitochondrial morphology, including wrinkling of mitochondria and increased membrane density [7]. In addition, Wang *et al*. [26] found that mitochondria contracted and ruptured significantly during ferroptosis triggered by iron overload. Mitochondria are the main source of ROS in cells. Therefore, ROS production in mitochondria may promote ferroptosis by promoting lipid peroxidation. Targeting mitochondria with antioxidants blocked doxorubicin-induced myocardial ferroptosis in mice, providing strong *in vivo* experimental evidence for a link between mitochondria and ferroptosis [26].

Mitochondrial iron metabolism also plays an important role in the regulation of ferroptosis. Heme oxygenase 1 (HO-1)-dependent heme degradation and labile iron overload may promote doxorubicin-induced ferroptosis and myocardial injury through HO-1-dependent mechanism in the mitochondria [27]. This is the first demonstration of a key mechanism for mitochondrial regulation of ferroptosis occurrence in a mouse model *in vivo*. Overexpression of mitochondrial ferritin (an iron storage protein located in the mitochondria) may inhibit erastin-induced ferroptosis by promoting iron storage in the mitochondria [28] (Fig. 1).

Mitochondria are an important organelle that provide energy to the cell. Ferroptosis is thought to be a new form of regulated cell death triggered by severe lipid peroxidation that is dependent on ROS production and iron overload. As a major site of iron utilization and a major regulator of oxidative metabolism, mitochondria are a major source of ROS and therefore play a role in ferroptosis. Indeed, ferroptosis is associated with severe impairment of mitochondrial morphology, bioenergetics and metabolism. So, it is understandable that ferroptosis may be closely linked to mitochondrial damage, but the potential causal relationship regarding whether ferroptosis causes mitochondrial damage or whether mitochondrial damage causes ferroptosis has yet to be explored. Of note, it has also been reported that ferroptosis is not linked to mitochondria [29]. However, it does not seem to be conclusive yet.

## 4. Defense Mechanism of Ferroptosis

The ferroptosis defense mechanism involves a cellular antioxidant system that directly neutralizes lipid peroxides. As will be described below, there are at least four ferroptosis defense systems each with unique subcellular localization (Fig. 1).

### 4.1 GPX4-GSH Antioxidant Defense System

There are multiple defense pathways against ferroptosis in cells, the main one of the defense pathways is mediated by GPX4 to inhibit the occurrence of ferroptosis by specifically catalyzing peroxidized lipids by GSH. GSH is a water-soluble tripeptide composed of glutamate acid, cysteine, and glycine amino acid residues. GSH is an important antioxidant in the human body, which removes free radicals. It can act as a cofactor of GPX4 in the reduction reaction of intracellular lipid hydroperoxide, thereby preventing the occurrence of ferroptosis. Knocking out GPX4 causes ferroptosis in cells [30]. GPX4 is the most important intracellular anti-lipid peroxidase and is an important regulator of ferroptosis. GPX4 through reducing lipid peroxidation protects cells from the threat of ferroptosis through a GSH-dependent manner [31]. In addition, solute carrier family 7 member 11 (SLC7A11) encodes a cysteine transporter protein that provides cells with cysteine, a key source of glutathione. In addition, SLC7A11 plays an inhibitory role against ferroptosis as an important component of Systemxc- among others [32]. Therefore, ferroptosis is defined as a novel concept of regulated cell death that is under constitutive control by GPX4 (Fig. 1).

### 4.2 FSP1-CoQH2 Antioxidant Defense System

Ferroptosis-suppressor-protein 1 (FSP1), which was previously thought to be involved in the induction of apoptosis [33], has a complex and somewhat controversial role in apoptosis [34, 35, 36, 37]. However, recent studies have shown that the nicotinamide adenine dinucleotide phosphate (NADPH)/FSP1/coenzyme Q (CoQ) axis acts as an independent system, together with the SLC7A11/GSH/GPX4 system, to protect cells from ferroptosis [38, 39]. FSP1 localizes to the plasma membrane and reduces CoQ to ubiquinol (CoQH2) by consuming NADPH [38, 39]. CoQH2 then inhibits ferroptosis by trapping lipophilic radicals [38, 39]. However, the mechanism involved in FSP1 as an antioxidant remains unclear. Although CoQ is mainly synthesized in the mitochondria, it has been detected in non-mitochondrial membranes. Thus, the pathway used by FSP1 to inhibit ferroptosis is unclear and the source of FSP1-mediated non-mitochondrial CoQ formation remains to be determined (Fig. 1).

### 4.3 DHODH-CoQH_2_ Antioxidant Defense System

A recent study revealed a local mitochondrial defense system mediated by dihydroorotate dehydrogenase (DHODH) that inhibits mitochondrial lipid peroxidation in the absence of mitochondrial GPX4 [40]. GPX4 inactivation causes significant lipid peroxidation, which in turn triggers ferroptosis, but not with significant mitochondrial lipid peroxidation. This suggests that GPX4 inactivation-induced ferroptosis is mainly triggered by non-mitochondrial lipid peroxidation. However, the results of the new study provide an explanation to the above-mentioned problem [40]. It was shown that DHODH, an enzyme involved in pyrimidine synthesis, reduces CoQ in the inner mitochondrial membrane to CoQH2 [40]. When GPX4 was dramatically inactivated, the cells neutralized lipid peroxidation and prevented mitochondria-triggered ferroptosis by significantly upregulating DHODH, leading to enhanced efficiency of CoQH2 production. Thus, inactivation of mitochondrial GPX4 and DHODH triggers sufficient mitochondrial lipid peroxidation and strongly induces ferroptosis [40]. Although mitochondrial GPX4 and DHODH can jointly inhibit mitochondrial lipid peroxidation through mutual compensation, this is not the case for GPX4 and FSP1 in the cytoplasm. Superficially, they cannot explain the lipid peroxides that accumulate in the inner mitochondrial membrane, because they are not in the same location. This suggests the importance of regional partitioning in ferroptosis defense [41, 42]. Further studies are needed to confirm this regionalized model in the regulation of ferroptosis and to explain some of the conflicting data obtained from previous studies. For example, cytoplasmic GPX4 was also found to be localized to the membrane gap of mitochondria. The abundance of cytoplasmic GPX4 in mitochondria and its potential role in inhibiting lipid peroxidation in mitochondria remains to be elucidated. And, another outstanding question concerns the potential role of other CoQH2-producing mitochondrial enzymes in the regulation of ferroptosis (Fig. 1).

### 4.4 GCH1-BH4 Antioxidant Defense System

Tetrahydrobiopterin (BH4) and its rate-limiting enzyme guanosine 5^′^-triphosphate cyclohydrolase 1 (GCH1) were recently identified as alternative ferroptosis defense systems independent of GPX4 [43, 44]. BH4 is a coenzyme for aromatic amino acid hydroxylases and other enzymes, and GCH1 mediates rate-limiting reactions in the BH4 biosynthetic pathway. BH4 is a potent radical-trapping antioxidant that promotes CoQH2 production and inhibits lipid peroxidation and ferroptosis [43, 44, 45]. It can also be regenerated by dihydrofolate reductase (DHFR), and thus, inactivation of DHFR significantly increases the vulnerability of cells to ferroptosis [45] In contrast, the subcellular localization of the GCH1-BH4 system in which it functions remains to be defined (Fig. 1).

## 5. Other Ferroptosis Regulatory Signals

Nuclear factor-erythroid 2-related factor 2 (NRF2) is a member of the transcription factor family of cap and collar leucine zipper structures. Physiologically, kelch-like ECH-associated protein 1 (KEAP1) binds to NRF2 in the cytoplasm to prevent NRF2 from entering the nucleus. When the cell suffers from the influx of ROS, KEAP1 will isolate from NRF2, and it in turn enters the nucleus and binds to the antioxidant response element (ARE) on the target gene promoter, facilitating the transcription of antioxidant genes, including ferritin expression, maintaining the cellular steady-state and redox system [46]. Furthermore, NRF2 transcription factors can regulate GSH homeostasis, mitochondrial function, and lipid metabolism, which are all associated with many molecular aspects of ferroptosis (Fig. 1) [47]. However, the exact mechanism of how NRF2 regulates ferroptosis remains unclear.

Tumor protein p53 (p53) is known to have a wide range and powerful functions. p53 functions as a transcription factor by activating or inhibiting the transcription of multiple downstream target genes. The role of these target genes includes newly discovered genes regulating ferroptosis. In 2015, Jiang *et al*. [48] revealed for the first time that p53 could inhibit tumor development by promoting cellular ferroptosis. The study revealed that p53 can be transcribed to inhibit SLC7A11 expression and promote ferroptosis (Fig. 1). Subsequently, Ou *et al*. [49] found that p53 could induce the expression of SAT1, and promotes the function of arachidonate 15-lipoxygenase (ALOX15), another member of the ALOX family, to enhance cell ferroptosis. In 2019, Chu *et al*. [50] demonstrated that the lipid oxidase arachidonate 12-lipoxygenase (ALOX12) is a key regulator of the occurrence of p53-dependent ferroptosis. This study indicates that ALOX12 is released when p53 downregulates SLC7A11 (Fig. 1). ALOX12 can oxidize the PUFAs of the cell membrane phospholipid leading to cell ferroptosis. In addition, Ou *et al*. [51] also found that p53 can regulate phosphoglycerate dehydrogenase (PHGDH) to inhibit serine synthesis and may affect GSH synthesis to promote ferroptosis. p53 promotes ferroptosis by inducing lncRNA plasmacytoma variant 1 (LncRNA PVT1) expression [52] or by direct binding to mitochondrial iron transporter solute carrier family 25 member 28 (SLC25A28) [53]. The above-mentioned findings provide strong evidence that supports the effects of p53 on ferroptosis. Together, despite the presence of a few counter examples [54], p53 in the vast majority of cases has been shown to promote cell ferroptosis. This may provide a new idea for the treatment of tumors, especially those with mutations in p53 [55, 56].

## 6. Ferroptosis and Oxidative Stress

Oxidative stress is one of the key pathogenic mechanisms of MIRI. Under physiological conditions, free radicals are components of the normal substance metabolism of the body and are the material basis for the maintenance of several important physiological functions. However, under pathological conditions, such as during MIRI, large amounts of ROS can be generated suddenly through the mitochondria, NADPH oxidase and other pathways [57]. ROS are considered harmful as by-products of metabolism and they can interact with cell membranes, DNA, macromolecular substances, and proteins, thus playing an important role in the pathophysiology of MIRI. Therefore, ROS are double-edged swords, on the one hand, they are important signaling molecules that regulate the structural and functional state of the vascular system, and on the other hand, they are also harmful substances during oxidative stress.

During MIRI, the excessive production of ROS affects the normal functioning of the mitochondria. Its main manifestations are the oxidation of mitochondrial lipids, the reduction of mitochondrial DNA copy number and the decrease of transcription [58, 59, 60]. Mitochondrial DNA is close to the mitochondrial respiratory chain, and it lacks protection similar to chromatin junctions, and has poor repair capacity for DNA damage. Therefore, in the context of MIRI, mitochondrial DNA becomes the main target of damage under the action of large amounts of ROS [58, 59, 60]. Furthermore, during IRI, ROS is a key factor in inducing mitochondrial permeability transition pore (mPTP) opening, which exacerbates MIRI by decoupling mitochondrial oxidative phosphorylation, halting ATP synthesis, and further promoting ROS production [57]. NADPH oxidase is a specialized functional enzyme that regulates ROS production. The mitochondrial respiratory chain and the NADPH oxidase (NOX) family are considered to be important sources of ROS [57]. During reperfusion, when O_2_ reaches the site of ischemia, it causes upregulation and activation of NADPH oxidases and subsequent production of excessive ROS [57].

Under pathological conditions such as during ischemia, the cardiomyocyte mitochondrial transport chain is decoupled, generating and releasing large amounts of free radicals [58, 59, 60]. Oxygen radicals contain unpaired electrons and are extremely unstable in nature, capable of attacking PUFAs on the mitochondrial membrane and triggering lipid peroxidation [61, 62]. Impaired mitochondrial function leads to impaired ATP synthesis and reduced energy required for mechanical work in the myocardium. The reason for this is that mitochondrial membranes contain more PUFAs than other membranes and are more sensitive to lipid peroxidation [61, 62]. During myocardial ischemia, ischemia and hypoxia caused by coronary artery constriction produce a large amount of oxygen free radicals [58, 59, 60]. This results in enhanced lipid peroxidation, membrane permeability and fluidity, which in turn leads to changes in myocardial function and structure, aggravating myocardial ischemic injury.

The abovementioned cellular events are consistent with ferroptosis manifestation and can be prevented by iron chelation and antioxidants. Indeed, iron chelation has been reported to be beneficial in some animal models of ischemia/reperfusion-related injury [63, 64]. It has been reported that some phenol-based molecules with antioxidants properties such as salvia miltiorrhiza, ginsenoside compound, and propofol can reduce oxidative stress levels during MIRI, but their role and potential impact on ferroptosis are unclear, while the importance of ferroptosis in MIRI has been given more or more intention recently [65, 66].

## 7. Ferroptosis and MIRI

From the first introduction of the concept of MIRI, it was confirmed that reperfusion causes irreversible necrosis of myocardial ultrastructure, leading to a decrease in cardiac function. However, the specific mechanisms involved in MIRI are not entirely clear. Oxidative stress is known to play an important role in MIRI. In contrast, there is a close relationship between ferroptosis and oxidative stress. With the progress of research, ferroptosis has been identified as a form of cell death closely related to oxidative stress in the pathogenesis of MIRI.

To reveal the specific mechanism of ferroptosis in IRI, Li *et al*. [67] found that ferrostatin-1 (Fer-1) improved the prognosis of heart transplantation by correlating the *in vivo* aseptic inflammatory response to IRI occurring after heart transplantation. They found that this mechanism of action was independent of necrosis, in which ferroptosis played an important role [67]. Most current studies investigating the role of ferroptosis in MIRI have focused on the ferric ion, lipid peroxidation, GPX4, and ferritin autophagy pathways.

### 7.1 Iron Plays an Important Role in MIRI

**Fig. 2.**
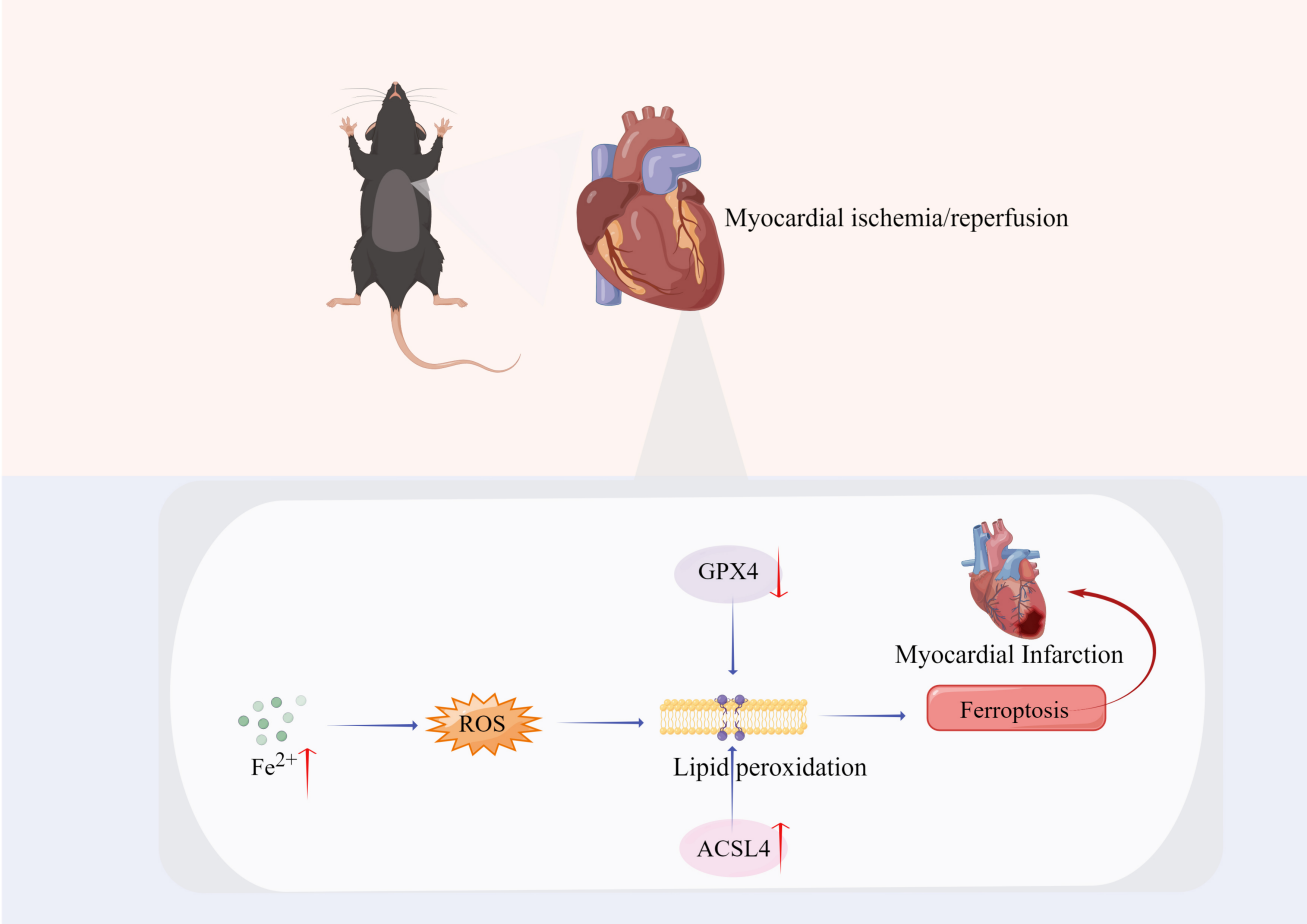
**Ferroptosis plays an important role in MIRI**. When mice suffer from myocardial ischemia/reperfusion, increased iron, cytoacidosis and intracellular environmental instability promotes the release of trivalent iron or sub-irons from iron-sulfur clusters and promote iron-mediated Fenton chemistry, producing a large number of free radicals. In the meantime, GPX4 decreased and ACSL4 increased during reperfusion. This induces lipid peroxidation production, which, subsequently leads to cardiomyocyte oxidative damage, causing ferroptosis in cardiomyocytes. Abbreviation used: MIRI, myocardial ischemia/reperfusion-related injury; ROS, reactive oxygen species; GPX4, glutathione peroxidase 4; ACSL4, long-chain acyl-CoA synthetase-4.

Iron, as a reactive element, plays an important role in redox reactions. Both iron overload and LIP may be risk factors for increased infarct size during reperfusion (Fig. 2). When ferritin is saturated, LIP remains present and participates in the Fenton reaction, generating OH^–^ [68]. This free radical is an important indicator of iron charge, which is highly active and therefore causes oxidative stress in various cells [69]. Alternatively, iron deposition increases in the surrounding infarct and non-infarct regions of a mouse myocardial infarction model [70]. Positive iron staining was further seen in non-cardiomyocytes surrounding the myocardial scar. In patients with acute ST-segment elevation myocardial infarction, iron deposition in cardiac tissue results in increased damage [71]. Consistent with this, a study using cardiac magnetic resonance imaging showed that after the patients had undergone percutaneous coronary intervention (PCI), researchers were surprised to find a correlation between adverse left ventricular remodeling and residual myocardial iron in patients [72]. This finding suggests that during MIRI, the ischemic phase may cause erythrocytolysis, leading to the local iron overload. This accumulation of iron produces an excess of ROS [73]. Despite ferritin synthesis during AMI, ferritin degradation produces the release of iron into the coronary blood flow [74]. Earlier studies have shown that relatively high concentrations of iron are mobilized into the coronary blood flow after prolonged ischemia [74, 75, 76]. Furthermore, excessive ROS causes an imbalance in redox homeostasis that impairs ferritin and iron-sulfur enzymes containing clusters [77].

### 7.2 Lipid Peroxidation is Involved in the MIRI Process

During ischemia/reperfusion, AA metabolism is triggered by the activation of calcium-dependent phospholipases due to intracellular calcium iron overload. AA produces large amounts of OH^–^and H_2_O_2_ through the action of cyclooxygenase and LOXs, both of which act on the cell membrane to further form lipid peroxides [78]. The production of lipid peroxides in turn accelerates AA metabolism, thus generating a large amount of ROS in AA metabolism, which further promotes the imbalance of AA metabolism, resulting in a vicious circle [78]. In addition, ACSL4 is a key enzyme that regulates lipid composition and plays a crucial role in the execution of ferroptosis. Another recent study has shown that ACSL4 is associated with MIRI. Increased ACSL4 protein expression was detected in animals suffering from MIRI [79]. Downregulation of ACSL4 inhibited ferroptosis occurrence, effectively protecting against myocardial injury [80]. Of note, a study showed that the ACSL4 and GPX4 protein levels in ischemic hearts did not significantly change during ischemia at different time points [81]. However, a gradual increase in ACSL4 protein levels occurred with increasing reperfusion time, which was accompanied with a gradual decrease in GPX4 protein levels in the cardiac tissue [81]. Similarly, in a study regarding intestinal IRI, it was found that a prolonged reperfusion time led to a decrease in GPX4 expression and an increase in arachidonic acid expression [82]. This study suggests that ferroptosis is more active at 30 minutes after reperfusion and less active at other moments of the pathology.

### 7.3 GPX4 is Involved in MIRI Regulation

In 2019, Park *et al*. [83], found that in a mouse myocardial infarction model, the GSH metabolic pathway was significantly downregulated in AMI. They revealed that the downregulation of GPX4 occurs at the transcriptional level. The authors also found that ferroptosis occurred in cardiomyocytes after inhibiting GPX4 expression *in vitro*. Feng *et al*. [81] found that in mouse myocardial ischemia/reperfusion model, the reperfusion simultaneously stimulates liproxstatin-1 (Lip-1), which can reduce the ischemic infarction area and improve mitochondrial structure and function. The relevant mechanism is one in which Lip-1 increases the content of mitochondrial antioxidant GPX4, reducing the production of ROS in the mitochondria. Thus, during MIRI, increased expression of GPX4 inhibits ferroptosis and mitigates myocardial injury. However, in the context of MIRI, most studies have only superficially concluded that ferroptosis in cardiomyocytes may be regulated by autophagy, while the underlying mechanisms of action are still poorly understood. Therefore, more in-depth studies in this area should be conducted in the future, in order to identify new breakthroughs in the treatment of MIRI.

## 8. Ferroptosis and Diabetes

Ndumele *et al*. [84] carried out an epidemiological investigation about the relationship of diabetic patients and cardiovascular disease. Surprisingly, the incidence of myocardial ischemia in diabetic patients was 2.45 to 2.99 times higher than that in non-diabetic patients [84]. In asymptomatic type 2 diabetes mellitus (T2DM) patients, the prevalence of asymptomatic myocardial ischemia was 20% to 30% [85]. Ischemic heart disease is the major cardiovascular complication and cause of death in diabetic patients. Recent studies by others and us show that the levels of ferroptosis increase in various organs in subjects with diabetes [86, 87], in particular in the hearts [88, 89, 90]. However, little is known regarding how ferroptosis may impact on the myocardial vulnerability to MIRI in diabetes.

### 8.1 Iron Deposition in T2DM

Ferroptosis is directly related to ferritin levels *in vivo* and epidemiological studies have revealed a potential association between excess iron stores in the body and T2DM [91, 92, 93]. Earlier studies have revealed an association between iron and T2DM in the development of insulin resistance [91, 94]. In addition, a meta-analysis found a positive association between organismal ferritin and the prevalence of T2DM, and the association was more pronounced in women [95]. There are also observational studies that have found an association between obesity and iron deficiency [96, 97]. Given that observational studies are susceptible to confounding factors and it is often difficult to determine causality, Wang *et al*. [98] further investigated the causal relationship between systemic iron status and T2DM by mendelian randomization analysis. This study revealed that elevated human iron levels are an important contributor in the development of T2DM [98]. Despite this, some studies have shown no association between body iron stores and the incidence of diabetes [99, 100]. However, it should not be ignored that obesity is also a major factor in the development of diabetes. Available evidence suggests that obese patients have a tendency to have higher hemoglobin and ferritin concentrations and lower transferrin saturation [96]. This suggests a strong association between iron and obesity. In addition, one study suggested the necessity of early monitoring and treatment of iron deficiency in overweight and obese individuals [96]. From the point of view of improving diabetes, it has been shown that improved insulin secretion and insulin sensitivity as well as better glycemic control can be observed after reducing the levels of body iron stores [101, 102].

### 8.2 Ferroptosis in T2DM and Metabolic Disorders

Disturbed lipid metabolism is an important pathophysiological process in diabetes mellitus In the diabetic heart, increased fatty acid uptake and decreased glucose utilization have been observed in animal models of diabetes and in type 2 diabetic patients [103, 104]. Peroxisome proliferator-activated receptor alpha (PPARα) is a transcription factor that can be present at high concentrations in the heart and is involved not only in the regulation of lipid metabolism and glucose homeostasis [105]. It has shown that PPARα activity is essential in the regulation of ferroptosis [106]. There are many other proteins that can be regulated either through PPARα or independently to predispose cells to ferroptosis. PPARα may be a key mechanism for regulating lipid peroxidation, a marker of ferroptosis-regulated cell death [107]. PPARα knockout mice undergo more severe iron accumulation and ferroptosis in the liver when fed a high iron diet [108]. The study further found that PPARα inhibited ferroptosis through GPX4 [108]. However, the exact mechanism of how PPARα is regulated in diabetic cardiomyopathy remains incompletely understood.

### 8.3 Impact of NRF2 on Ferroptosis in Diabetes

**Fig. 3.**
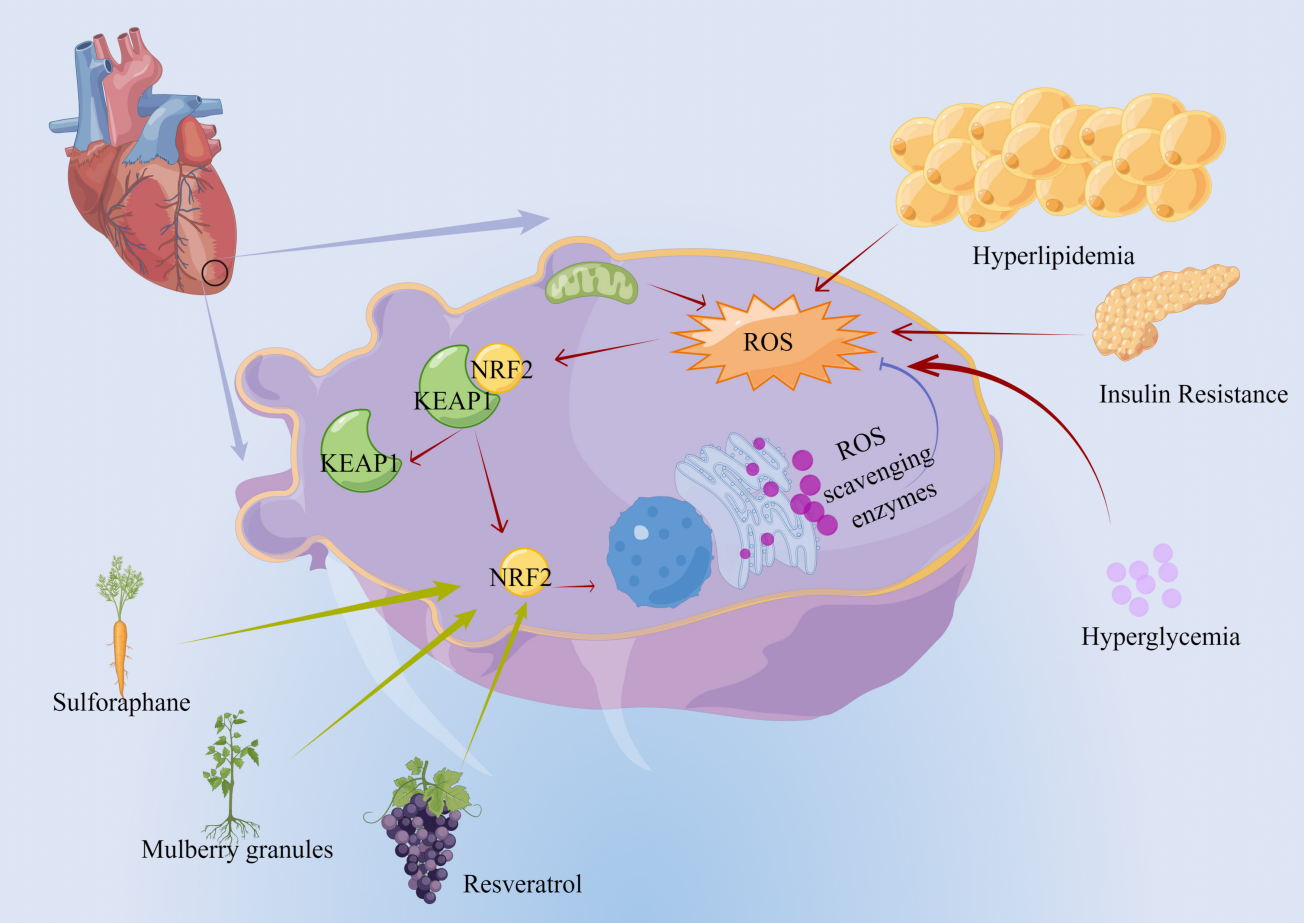
**The impact of NRF2 on ferroptosis in diabetes**. Chronic hyperlipidemia, insulin resistance, and hyperglycemia induce increased intracellular ROS and elevated intracellular oxidative stress. In response, KEAP1 is separated from NRF2, which enters the nucleus and binds to the ARE on the target gene promoter to promote transcription of antioxidant genes, including ROS scavenging enzymes, maintaining cellular redox system. Some natural bioactivities, such as sulforaphane, mulberry granules, and resveratrol, can induce NRF2 expression, increase intracellular antioxidant and inhibit ROS. Abbreviation used: ROS, reactive oxygen species; NRF2, nuclear factor erythroid 2-related factor 2; KEAP1, kelch-like ECH-associated protein 1; ARE, antioxidant response element.

Oxidative stress and impaired antioxidant systems underlie the pathogenesis of T2DM. NRF2 has been reported to play an important role in diabetic cardiomyopathy [46, 47]. Activation of NRF2 to inhibit ferroptosis may be a potential therapeutic target for T2DM in animal models. It has also been shown that the NRF2 signaling pathway is a key mechanism in diabetic MIRI, limiting ferroptosis by regulating iron metabolic homeostasis, and activation of NRF2 signaling pathway may alleviate diabetic MIRI to some extent [109, 110]. Some traditional Chinese medicines as well as natural active ingredients have been shown to be activators of NRF2 and have protective effects against diabetic myocardial MIRI [109, 110, 111] (Fig. 3). However, how the activation of NRF2 alters ferritin formation during the pathogenesis and progression of diabetic cardiomyopathy (DCM) is not clear. Enhancement of endogenous NRF2 may be an effective strategy to provide prevention and treatment for diabetic MIRI. However, there is increasing evidence that NRF2 also has adverse aspects in cardiovascular disease. Although further activation of the NRF2 pathway is beneficial to improve the prognosis of diabetes mellitus and its complications, it may even be applied to the treatment of clinically relevant diseases. However, the breakthrough finding is that NRF2 promotes cardiac injury when myocardial autophagy is impaired in pressure-overloaded hearts [112]. In fact, the “dual effect” of NRF2 has been confirmed by many studies [112, 113]. This will undoubtedly challenge the application of NRF2 agonists in the clinical treatment of diabetes. But at the same time, before carefully weighing the results of preclinical research and providing objective analysis, scientific research on NRF2 agonists may be applied to diabetes patients. Of note, current evidence suggests that diabetic patients suffering from chronic complications are highly likely to benefit from NRF2 agonist application [109, 110, 111]. Objectively speaking, whether the NRF2 pathway is equally protective in other unexplained chronic complications of diabetes or plays a “dual role” remains to be studied in the future.

## 9. Targeting Ferroptosis in MIRI Therapy

For the treatment of myocardial infarction, the most important priority is timely initiation of percutaneous coronary angioplasty to restore blood flow. However, there are multiple mechanisms that can trigger or exacerbate post-ischemic myocardial reperfusion injury, while the exact mechanisms and in particular their interplay during MIRI remain unknown. There is growing evidence indicating that ferroptosis and oxidative stress respectively and in particular their combination is major drivers MIRI, and thus could be promising therapeutic targets in an effort to reduce infarct size. There are a number of drugs that have been reported to inhibit ferroptosis and reduce MIRI [63, 64, 81, 114, 115].

As mentioned above, ferroptosis plays an important role in MIRI as well as in diabetes. Targeting MIRI therapy by inhibiting ferroptosis provides an opportunity. Deferoxamine (DFO) is a trivalent iron complex synthetic iron poisoning antidote. It protects cardiomyocytes by binding labile iron and preventing the formation of ROS from the Fenton reaction by reactive iron [63, 64]. DFO may reduce oxidative stress in cardiomyocytes after the onset of ischemia [114, 115]. Lip-1 is a lipid peroxide radical scavenger and a potent inhibitor of ferroptosis. Lip-1 reduces myocardial infarct size, decreases ROS, and protects the structural integrity of mitochondria after ischemia [81]. Fer-1 is a common ferroptosis inhibitor. It has been found to reduce myocardial reperfusion-induced lipid peroxidation and acts as a myocardial protector [116, 117].

### 9.1 Ferroptosis as a Major Determinant of Myocardial Susceptibility to IRI in Diabetes

Our most recent study showed that the post-ischemic myocardial infarct size in diabetic mice was smaller than that in non-diabetic control mice at 1 week of diabetes that was simultaneous with reduced cardiac levels of ferroptosis, while the post-ischemic myocardial infarct size at 5 weeks of diabetes was significantly greater than that in the non-diabetic control, which was associated with a significant increase in cardiac ferroptosis at 5 weeks of diabetes [89]. Treatment with the antioxidant N-acetyl-L-cysteine (NAC) significantly attenuated post-ischemic ferroptosis as well as oxidative stress and reduced infarct size at weeks of diabetes, while the application of erastin, a ferroptosis inducer, reversed the cardioprotective effects mediated by NAC therapy [89]. These findings are indicative that increased oxidative stress and ferroptosis are the major factors attributable to increased vulnerability to MIRI in diabetes and that attenuation of ferroptosis represents a mechanism whereby NAC confers cardioprotection against MIRI in diabetes [89]. The notion that ferroptosis may function as a major determinant of myocardial susceptibility to IRI is further supported by the findings that inhibiting Rev-erbα-mediated ferroptosis reduced myocardial susceptibility to IRI in type 2 diabetic mice [118] and that blockade of cardiac connexin 43 overexpression-mediated enhancement of ferroptosis attenuated MIRI in type 1 diabetic mice at a relatively late stage of diabetes [90].

### 9.2 Signaling Molecules that may Determine Cellular Susceptibility to Ferroptosis also Determine Myocardial Susceptibility to IRI in Diabetes

In most situations, GPX4 determines the susceptibility to ferroptosis, and its reduction may result in ferroptosis even if iron homeostasis is physiologically maintained [119, 120]. A recent study confirmed the determinant role of GPX4 in MIRI [121]. Furthermore, the finding that HO-1 upregulation in response to hypoxia/reoxygenation may induce iron overload and subsequently enhance ferroptosis susceptibility, which would minimize the cardioprotection potential of HO-1 during IRI [119]. HO-1 could catalyze the degradation of heme to produce Fe^2+^ and causes iron overload in the endoplasmic reticulum and increases the susceptibility to ferroptosis but still somehow plays a cardioprotective role against MIRI through reducing oxidative stress via biliverdin and carbon monoxide. Therefore, joint therapy targeting ferroptosis susceptibility can maximize the cardioprotection of HO-1. The intake of PUFAs has been believed to be beneficial for heart function for decades, but it was found that this might have been overstated in some circumstances recently [122, 123]. Ma* et al.*’s [124] latest data focused on the ischemia phase of ischemia/reperfusion demonstrating unequivocally that induction of ALOX15 triggers oxidization of PUFA-phospholipids, leading to cardiomyocyte ferroptosis. The author believes this to be a priming signal that augments robust oxidative damage and increases the susceptibility of ferroptosis in the reperfusion phase. Another study further found that peroxisomes contribute to ferroptosis through the synthesis of polyunsaturated ether phospholipids (PUFA-ePLs), an understudied lipid class that provide substrates for lipid peroxidation, contributing to heightened ferroptosis sensitivity in cardiomyocytes [125]. Given the above, when therapeutics target MIRI, attention should be paid to whether it accidentally triggers the susceptibility of ferroptosis in cardiomyocytes. If this does occur, concomitant therapy targeting ferroptosis especially maintaining the level and function of GPX4 is a good way to enhance the therapeutic effectiveness.

## 10. Conclusions

Current studies have shown that inhibition of ferroptosis during myocardial ischemia/reperfusion can attenuate post-ischemic myocardial reperfusion injury. However, questions regarding whether or not ferroptosis begins early during myocardial ischemia/reperfusion prior to the occurrence of other forms of programmed cell deaths, or vice versa, is it be triggered by other programmed cell deaths remain unanswered. Lipid peroxidation is a hallmark and central to ferroptosis, and lipid peroxidation induces the onset of ferroptosis. It follows that ferroptosis and apoptosis could be regulated at least in part via a similar or the same mediator during MIRI. An in depth mechanistic study about the regulation of ferroptosis and in particular its potential interplay with other forms of programmed cell death is critical to the treatment or prevention of MIRI especially MIRI in subjects with diabetes whose susceptibility to ischemic insult is increased, given inhibitors and inducers of ferroptosis as currently used in animal models still have a long way to go before being used clinically. Although numerous research articles and reviews have elucidated and summarized the regulation and molecular metabolic mechanisms of ferroptosis, there are still some challenges. For basic research, one of the unsolved questions is what is the ultimate executor of ferroptosis after lipid peroxidation? Answering this question may help identify other markers of ferroptosis and distinguish it from other forms of regulatory cell death [125, 126, 127]. For further clinical applications, there is no biomarker that can be used to specifically to diagnose ferroptosis [21, 126]. In addition, the timing, dosage, form of administration for ferroptosis inducers or inhibitors, and application duration remain to be standardized. Evaluation of the clinical safety and efficacy is also lacking. More preclinical and clinical trials are needed in the future to validate the role of ferroptosis, laying the foundation for the development of drugs for treating human diseases.

## Abbreviations

MIRI, myocardial ischemia/reperfusion-related injury; PCI, percutaneous coronary intervention; CABG, coronary artery bypass grafting; ROS, reactive oxygen species; AMI, acute myocardial infarction; GSH, glutathione; GPX4, glutathione peroxidase 4; PUFAs, polyunsaturated fatty acids; H_2_O_2_, hydrogen peroxide; NCOA4, nuclear receptor coactivator 4; LIP, labile iron pool; PE, phosphatidylethanolamine; LPCAT3, lysophosphatidylcholine acyltransferase 3; ACSL4, long-chain acyl-CoA synthetase-4; LOXs, lipoxygenase; AA, arachidonic acid; AdA, adrenic acid; HO-1, heme oxygenase 1; SLC7A11, solute carrier family 7 member 11; FSP1, ferroptosis-suppressor-protein 1; NADPH, nicotinamide adenine dinucleotide phosphate; CoQ, coenzyme Q; DHODH, dihydroorotate dehydrogenase; BH4, tetrahydrobiopterin; GCH1, guanosine 5^′^-triphosphate cyclohydrolase 1; DHFR, dihydrofolate reductase; NRF2, nuclear factor erythroid 2-related factor 2; KEAP1, kelch-like ECH-associated protein1; ARE antioxidant response element; ALOX12, arachidonate 12-lipoxygenase; PHGDH, phosphoglycerate dehydrogenase; LncRNA PVT1, lncRNA plasmacytoma variant 1; SLC25A28, solute carrier family 25 member 28; mPTP, mitochondrial permeability transition pore; T2DM, type 2 diabetes mellitus; PPARα, peroxisome proliferator-activated receptor alpha; DFO, desferrioxamine; Fer-1, ferrostatin-1; Lip-1, liproxstatin- 1; NAC, N-acetyl-L-cysteine; Bcl-2, member of the B cell lymphoma 2; RIP1, receptor interaction protein kinase 1; RIP3 receptor interaction protein kinase 3; mTOR, the mammalian target of rapamycin; Bax, Bcl-2 associated protein X; LC3, microtubule-associated protein 1 light chain 3; ATG5, autophagy-related 5; ATG7, autophagy-related 7; TFR1, transferrin receptor 1; SLC3A2, solute carrier family 3, member 2; Mitoferrin, mitochondrial ferritin; GPX4mito, mitochondrial GPX4; CoQ10, coenzyme Q10; CoQH2, ubiquinol; p53, tumor protein p53.
